# Supplementary material for: Scaling growth rates for perovskite oxide virtual substrates on silicon
Source: Nat Commun. 2019 Jun 5;10:2464. doi: 10.1038/s41467-019-10273-2 (PMC6549169; doi:10.1038/s41467-019-10273-2)
Supplement: Supplementary file 1 — Supplementary Information [file 41467_2019_10273_MOESM1_ESM.pdf]

Supplementary Information

Scaling growth rates for perovskite oxide virtual substrates on silicon

Jason Lapano[1], Matthew Brahlek[#1], Lei Zhang[2], Joseph Roth[1], Alexej Pogrebnyakov[1], Roman Engel-Herbert[1,3,4]*

[1]Department of Materials Science and Engineering, Pennsylvania State University, University Park, Pennsylvania 16802, U.S.A.

[2]Department of Materials Science and Engineering, University of California, Berkeley, California 94720, U.S.A.

[3]Department of Physics, Pennsylvania State University, University Park, Pennsylvania 16802, U.S.A.

[4]Department of Chemistry, Pennsylvania State University, University Park, Pennsylvania 16802, U.S.A.

[#] Current address: Materials Science and Technology, Oak Ridge National Lab, Oak Ridge, Tennessee, 37831, U.S.A.

*Correspondence should be addressed to rue2@psu.edu

Supplementary Fig. 1| Intrinsic $SrTiO_3$ film lattice parameter reported for different growth rates using scalable oxide thin film growth techniques: pulsed laser deposition (PLD) [1–4], sputtering [5–9], metal-organic chemical vapor deposition (MOCVD) [10–13], atomic layer deposition (ALD) [14–16], molecular beam epitaxy (MBE) [17–20], and hybrid molecular beam epitaxy (*h*MBE) [21–23]. In all cases except *h*MBE the degree of film nonstoichiometry increases with growth rate. The number close to data points shown refer to references detailed in Supplemental Figure 1. The defect concentration δ of Sr-rich ($Sr_{1+\delta}TiO_3$) and Ti-rich ($Sr_{1-\delta}TiO_3$) films due to non-stoichiometric growth condition was determined from the intrinsic film lattice parameter expansion using a calibration curve given in Supplemental Figure 2.

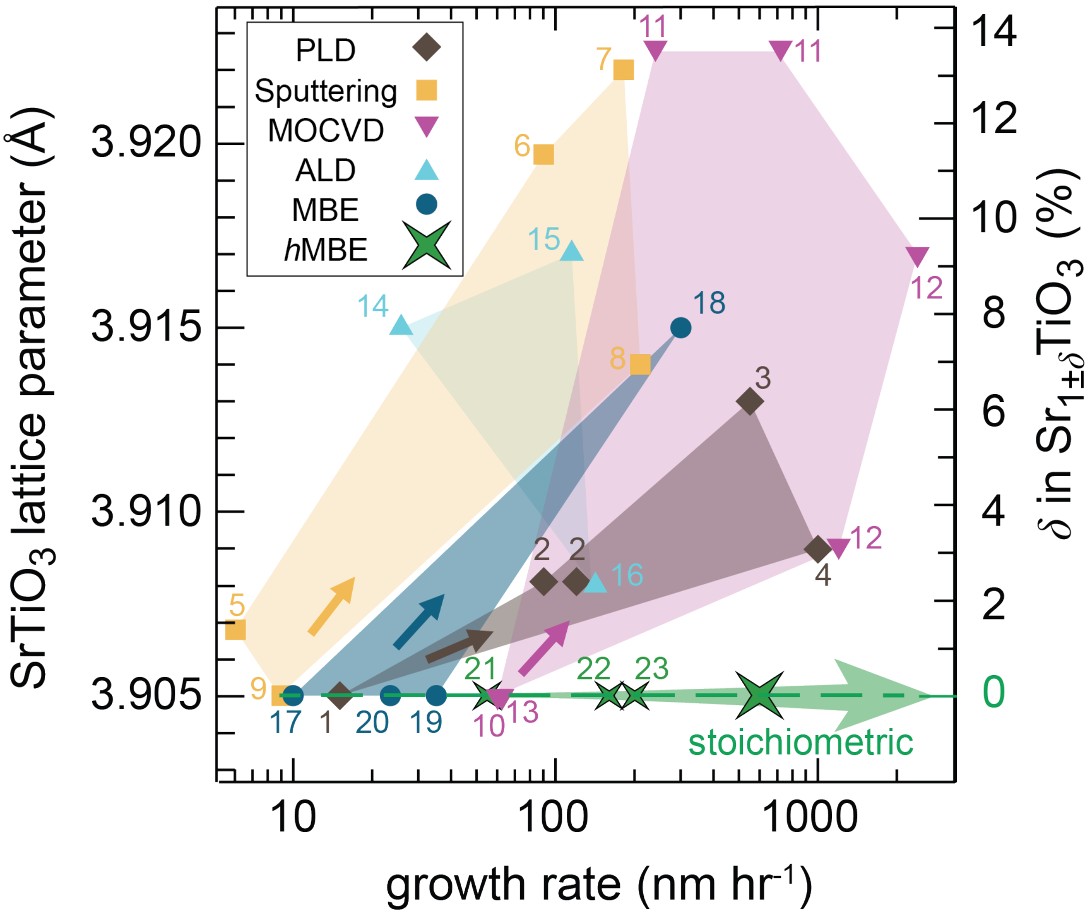

Supplementary Fig. 2| Intrinsic lattice parameter of homoepitaxial $SrTiO_3$ thin films grown by molecular beam epitaxy using experimental data from shutter deposition and co-deposition with different Sr content from Ref. 19 and data set from Ref. 24. The out-of-plane lattice parameter taken from Refs. 19 and 24 were converted to intrinsic film lattice parameter using a Poisson ratio for $SrTiO_3$ of 0.244[25]. The difference in the lattice parameter for Sr rich films were attributed to a different degree of SrO planar fault orientation. The linear interpolation was used to link lattice parameter reported for $SrTiO_3$ in Refs. 1-23 to their degree of nonstoichiometry.

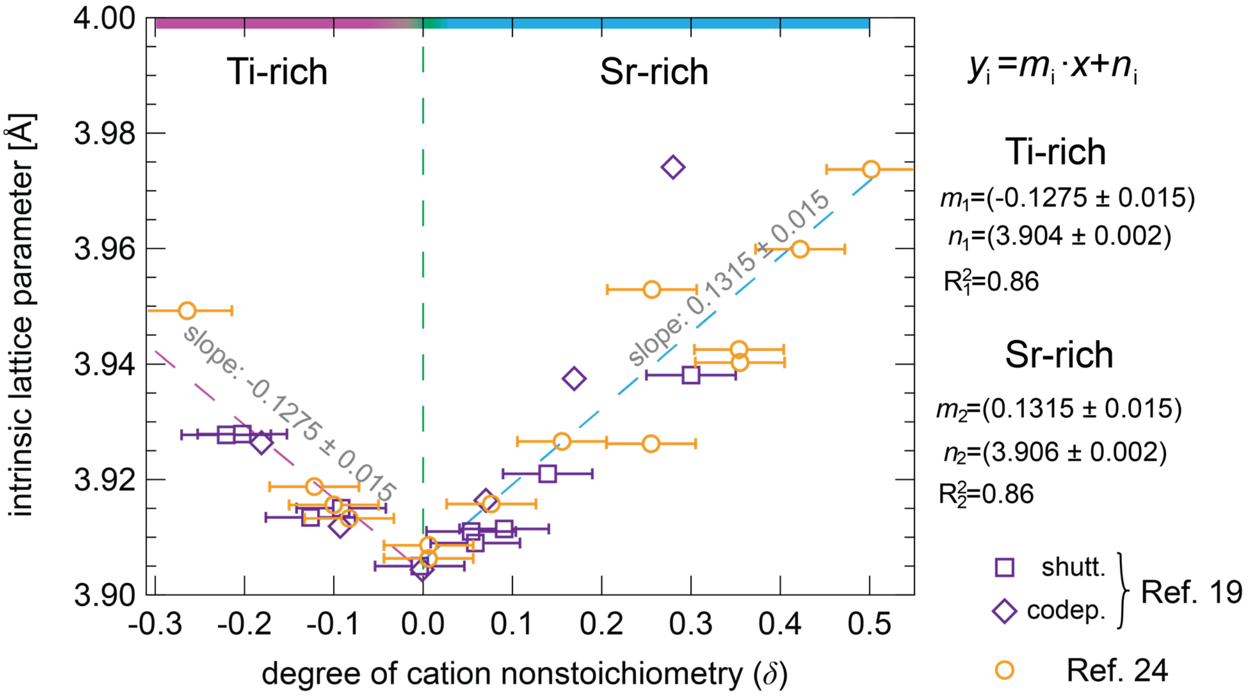

Supplementary Fig. 3| Reflection high-energy electron diffraction (RHEED) images during mapping of the growth window for a Sr flux of 2.5×10^{13} cm^{-2} s^{-1}, taken along the <100> and <110> azimuth of $SrTiO_3$. The stoichiometric conditions were found for a TTIP gas inlet pressure between 57 and 69 mTorr, which is converted to a TTIP beam equivalent pressure p_{TTIP} of 1.06×10^{-6} Torr and 1.77×10^{-6} Torr, respectively, using the calibration curve of beam equivalent pressure and gas inlet pressure shown in Supplementary Fig. 4.

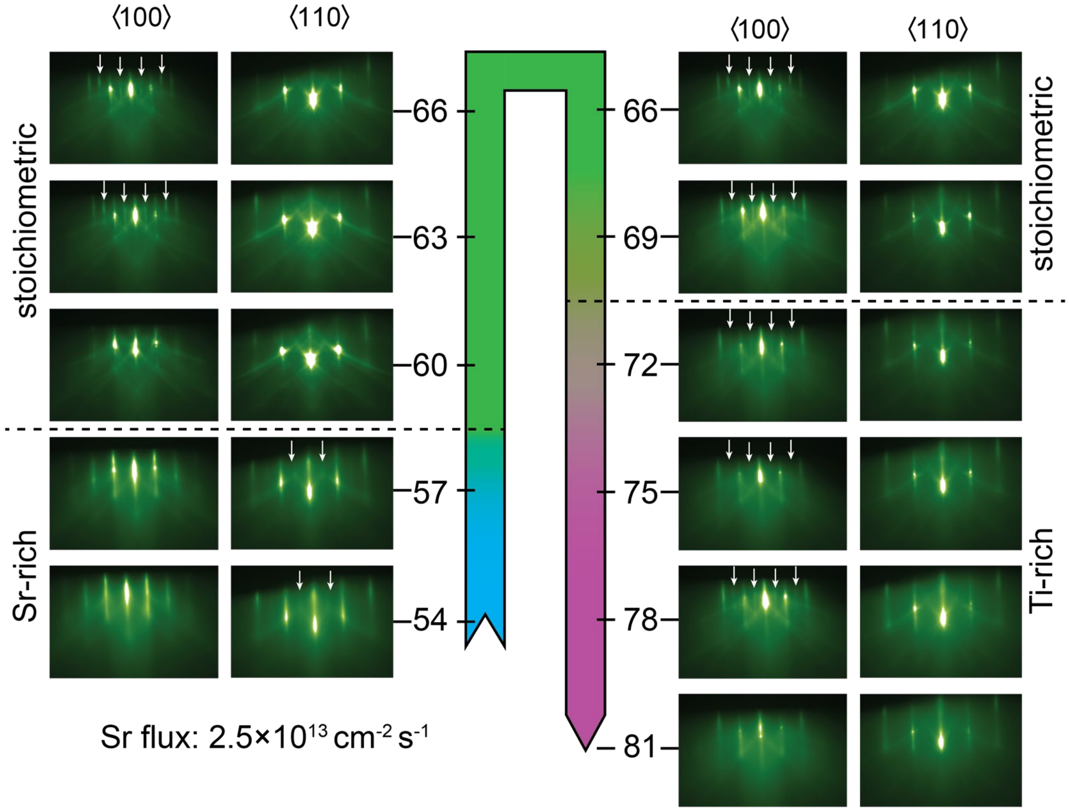

Supplementary Fig. 4| The calibration curve of titanium tetraisopropoxide (TTIP) beam equivalent pressure p_{TTIP} and gas inlet pressure. The data set was fit using a second order polynomic expression. A good agreement was achieved with an R^2 value of 0.99.

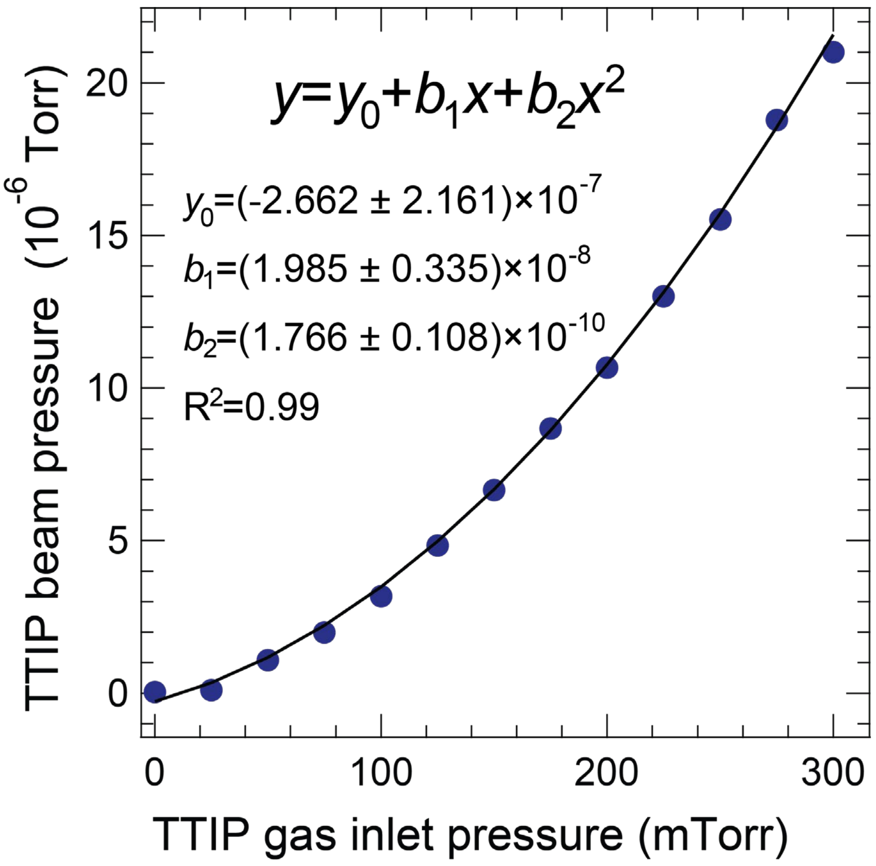

Supplementary Fig. 5| Growth rate extracted from GenX[26] fit of the X-ray data shown in Supplementary Fig. 7 for $SrTiO_3$ films grown on LSAT as a function of Sr flux measured by the quartz crystal monitor at sample position in units of $10^{13}cm^{-2}\,s^{-1}$ of Sr atoms. The relation can be approximated by a linear function, the fit has an R^2 value of 0.94. For Sr flux values exceeding 2.00×10^{14} $cm^{-2}\,s^{-1}$ the effusion cell has been found to become rather unstable with sizeable flux drifts of about 4% per hour resulting in a much smaller growth rate than expected from Sr flux calibrations using the quartz crystal monitor prior to the growth. The high Sr flux rates needed to demonstrate a growth rate of 600 nm hr^{-1} were generated from two Sr effusion cells operated in tandem. The significantly lower flux compared to the growth rate determined from film thickness measurements detailed in Supplementary Fig. 7 were attributed to a different tooling factor of the quartz crystal monitor.

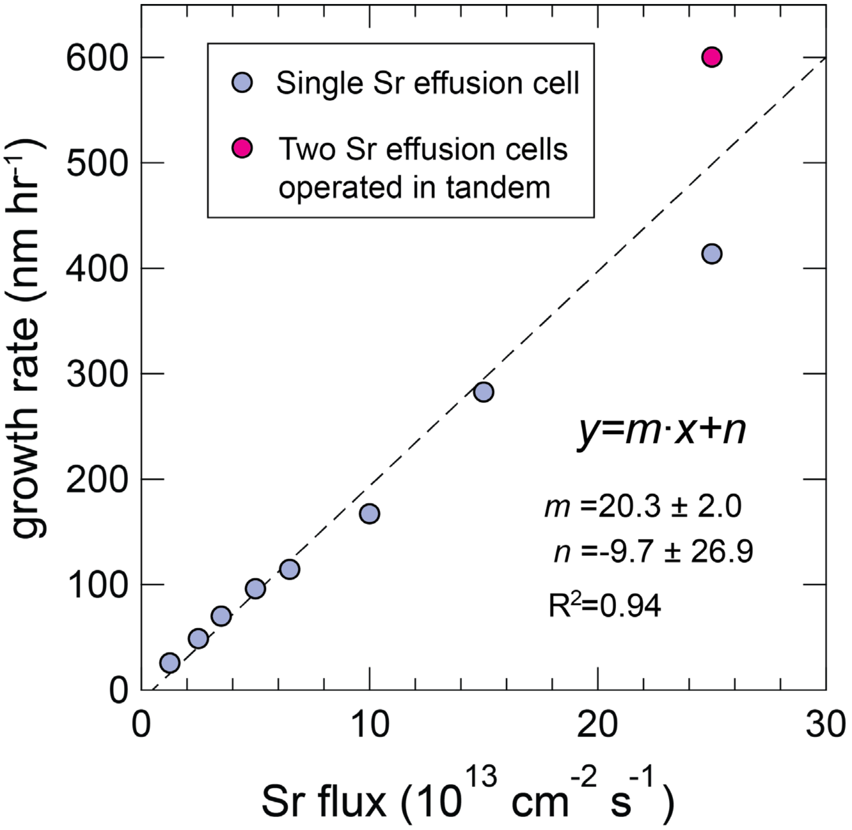

Supplementary Fig. 6| AFM images of nominally 45-nm-thick $SrTiO_3$ films grown on LSAT at varying growth rates α (top). All samples exhibit surface roughness of less than 1 nm. Samples grown at fluxes above 2.5×10^{13} Sr atoms cm^{-2} s^{-1} show step and terrace morphology. AFM images of nonstoichiometric films are shown (bottom). Defects of ~ 0.2 μm in size are seen on the Sr-rich sample, while the Ti-rich sample exhibited a smooth, featureless surface. All scale bars are 250 nm.

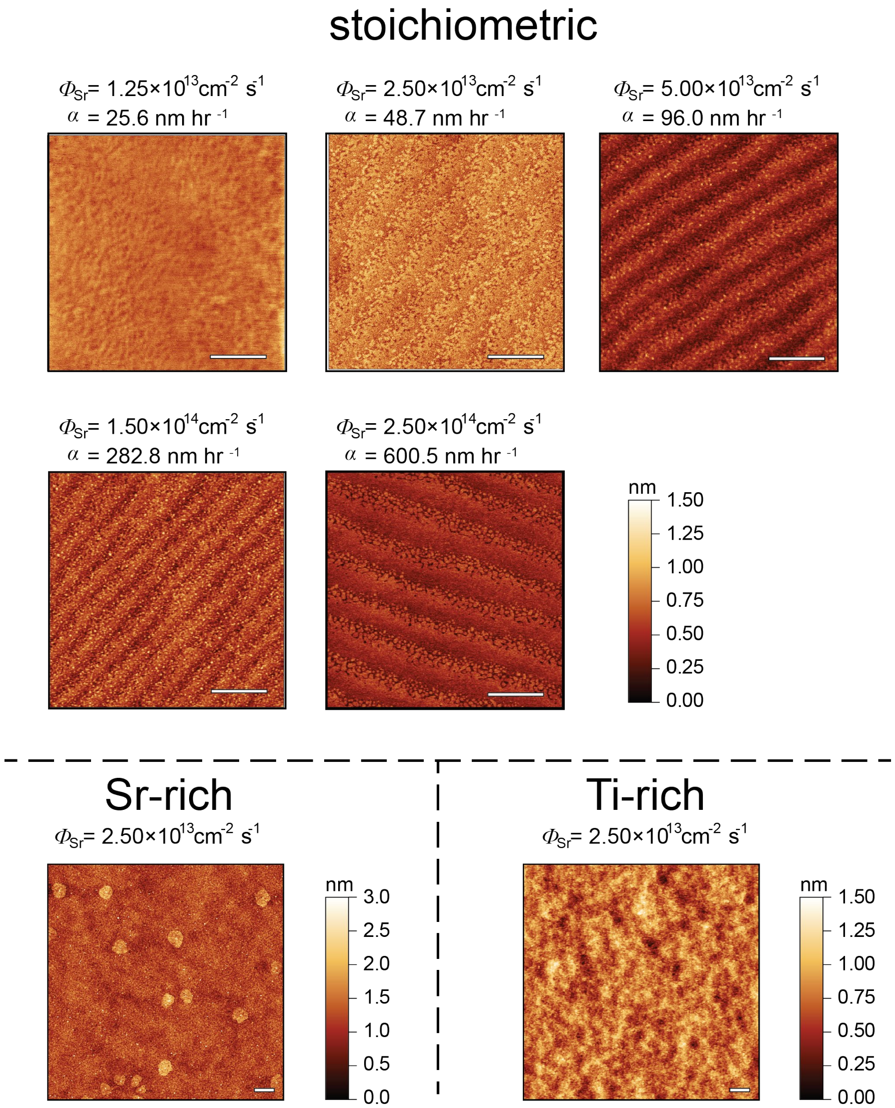

Supplementary Fig. 7| X-ray diffraction scans and fits obtained from GenX[26] for the growth rate scaling of $SrTiO_3$ films grown on LSAT. Excellent comparison of experimental data and fits were obtained. The film growth rate α was obtained from the film thickness determined from the von Laue thickness fringes and the growth time τ. Sizeable deviation from the expected out-of-plane lattice parameter was observed for the film grown at a growth rate of 413.7 nm hr^{-1}, attributed to the drift of a single Sr effusion cell operated at fluxes higher than 2.00×10^{14} cm^{-2} s^{-1}.

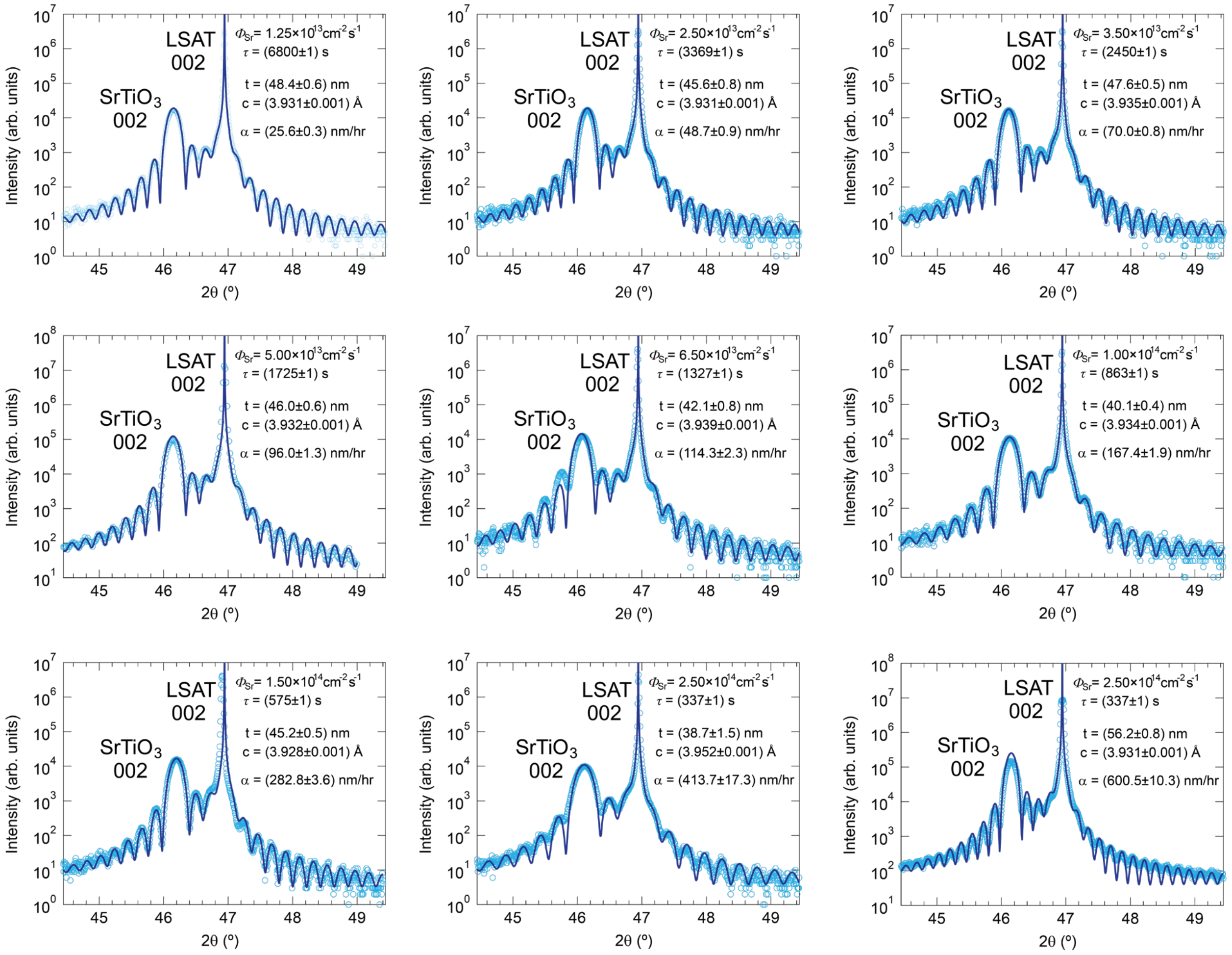

Supplementary Fig. 8| Wafer scale metrology of the metamorphic buffer layer $SrTiO_3$ on Si obtained from spectroscopic ellipsometry. Phase difference Δ and amplitude ratio Ψ were collected in the spectral range of 0.75 eV to 3.0 eV collected at 25 locations across the wafer. The thickness values of the individual layers, namely amorphous silicon oxide (*a*-SiO_x) interlayer thickness, $SrTiO_3$ film and $SrTiO_3$ surface layer thickness were extracted employing a least squares Levenberg-Marquardt regression algorithm using optical models of the Si substrate, *a*-SiO_x, $SrTiO_3$ and a $SrTiO_3$ surface layer to account for the film surface roughness using the Bruggeman effective medium approximation.

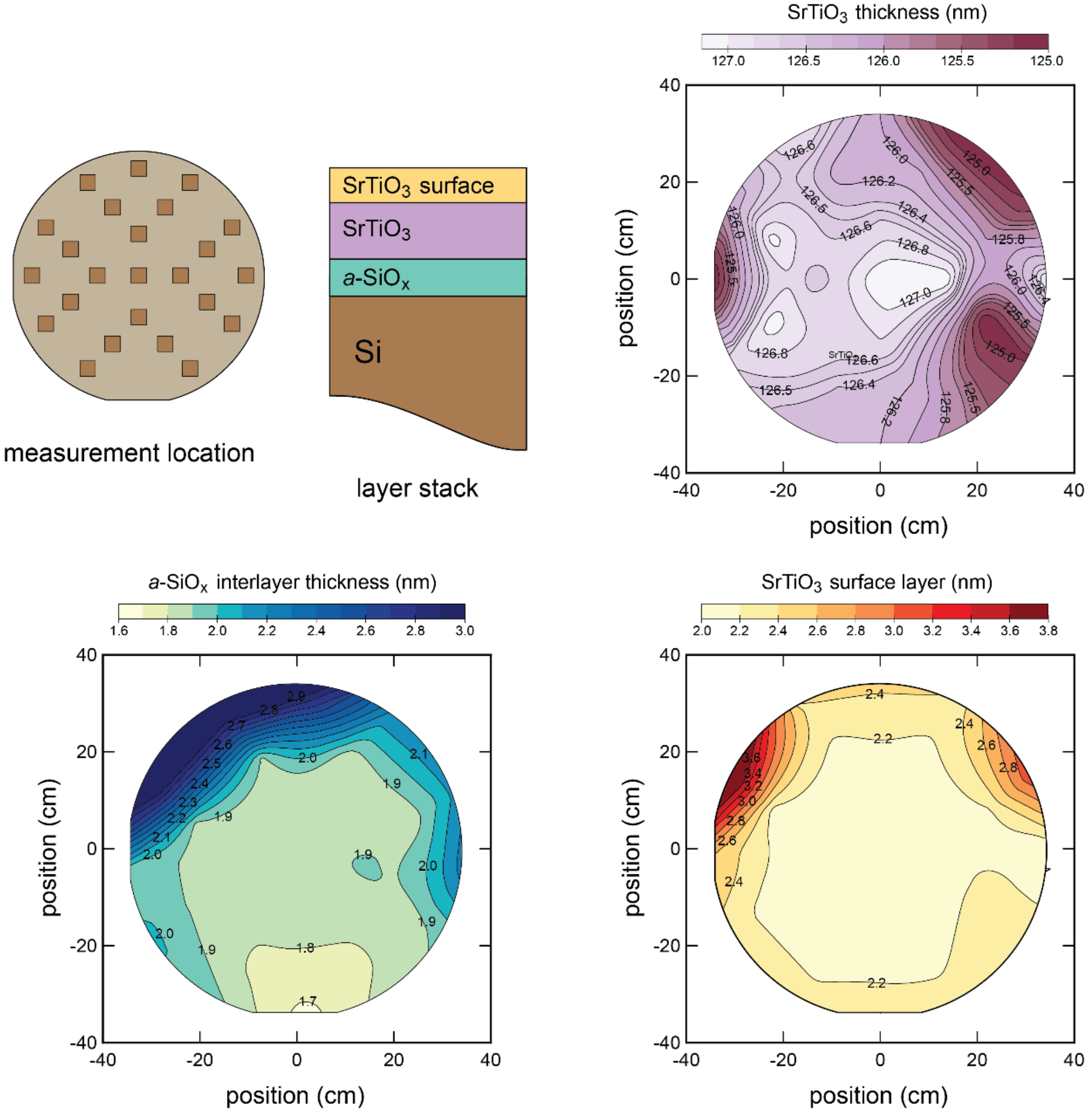

Supplementary Fig. 9| **a,** Cropped HAADF-STEM image of the Sr-rich region of the calibration sample shown in Fig. 2. The linear strain maps of the image are shown in **b, c.** Areas of high strain highlight the location of the horizontal and vertical Ruddeslden-Popper (RP). Scale bars are 3 nm. **d,** A composite of the strain components overlaying the original image, highlights the exact location of each RP fault.

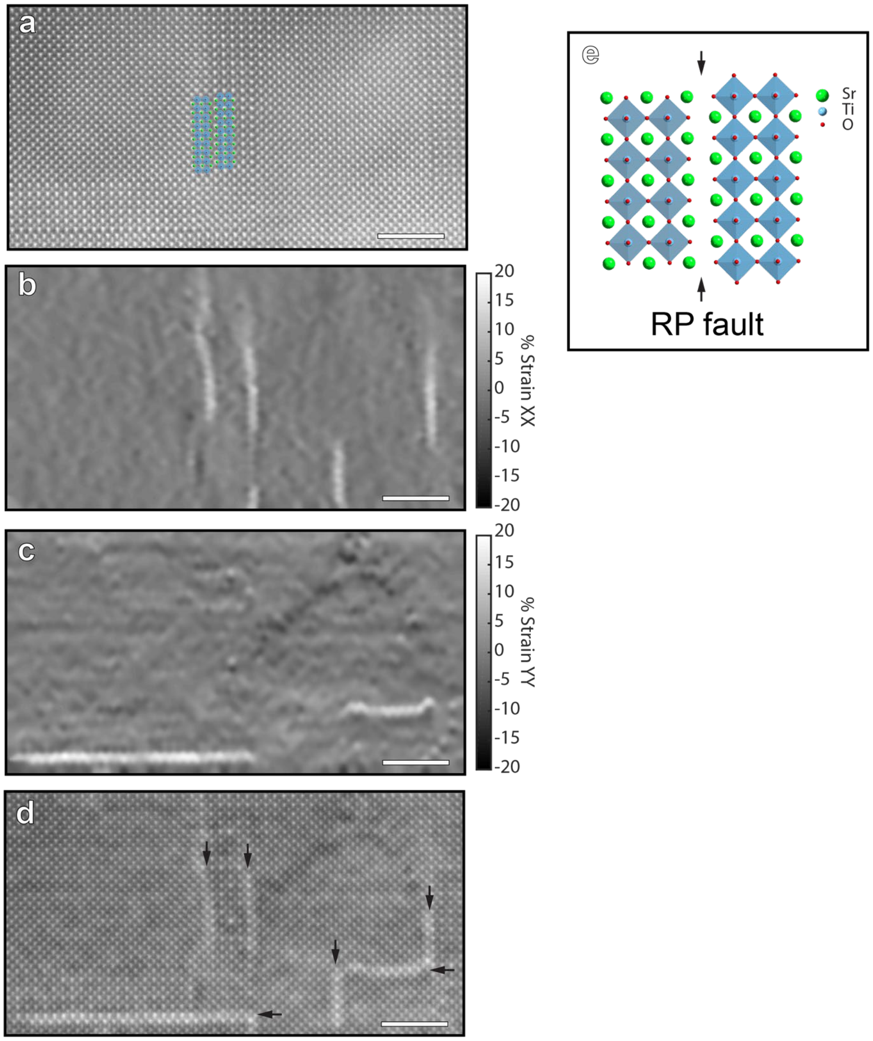

Supplementary Fig. 10| **a**, HAADF-STEM imaging of an un-annealed $SrTiO_3$ film grown on 3" silicon at 240 nm hr^{-1}. Low defect, single crystalline film was found at all observed areas, in agreement with x-ray results. Defects were found more closely concentrated near the interfacial region, and disappeared in the bulk of the film. The surface roughening is due to ion beam damage induced during sample preparation process. **b-g**, HAADF and energy dispersive x-ray spectroscopy (EDSX) imaging taken at the interface of the sample. All scale bars are 4 nm.

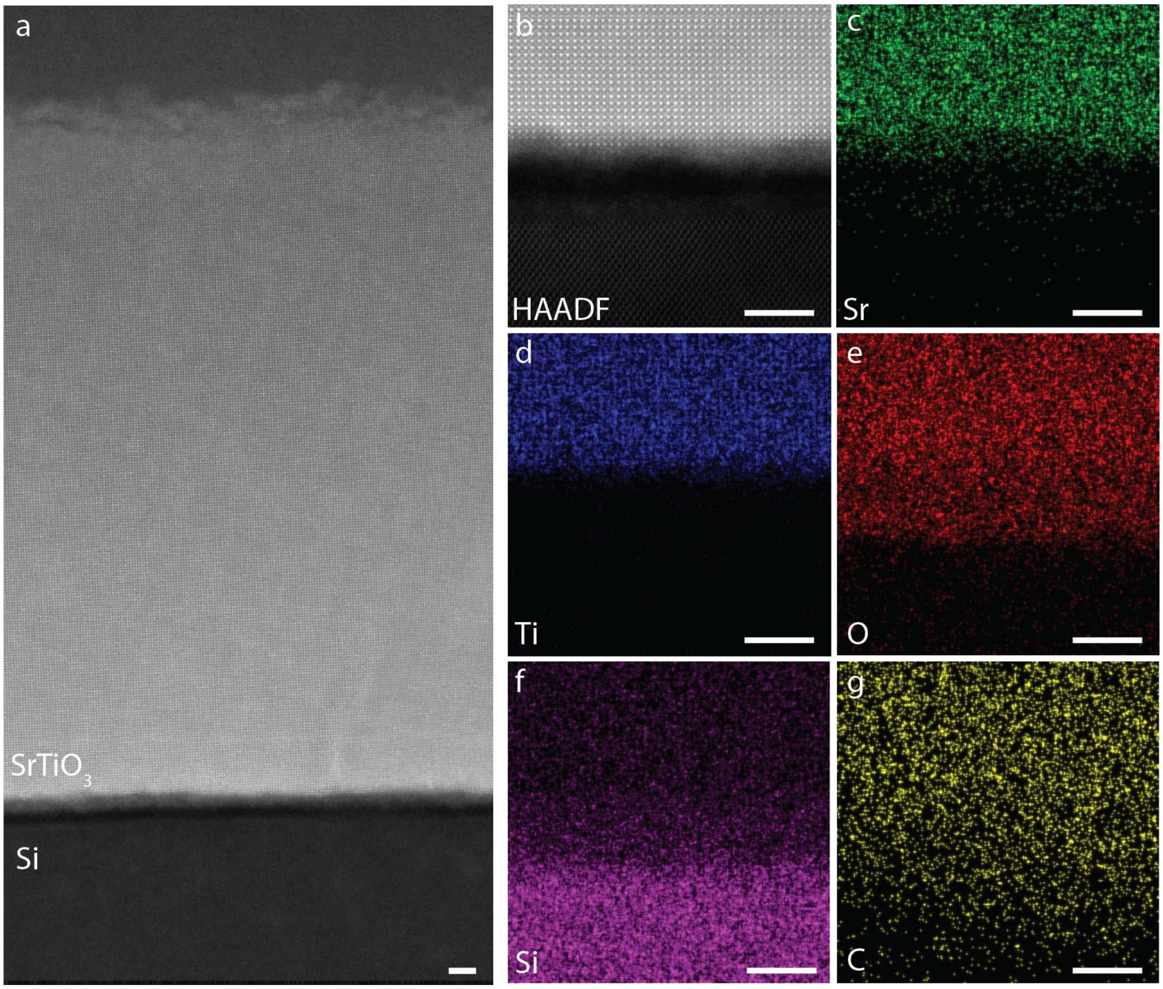

Supplementary Fig. 11| Secondary ion mass spectrometry (SIMS) of $SrTiO_3$ on silicon is obtained using a PHI nano TOF. A primary 30 keV Bi_3^+ ion beam was rastered over a 100 x 100 µm area. The etch rate is approximately 3 nm $cycle^{-1}$. Profile covers approximately 85 nm, and begins after the first 75 nm of film has been etched away. The small increase in the carbon signal at the start of the profile is due to adsorption of background gas species in the analytical chamber. A small carbon signal slightly above the detection limit is present throughout the film. The carbon concentration is estimate this to be in the mid-10^{17} atoms cm^{-3} based on previous studies into carbon incorporation of films growth by *h*MBE[27]. Carbon concentration increases at the interface due to lower cracking efficiency of the carbon-containing precursor at lower temperatures during deposition of the buffer layer.

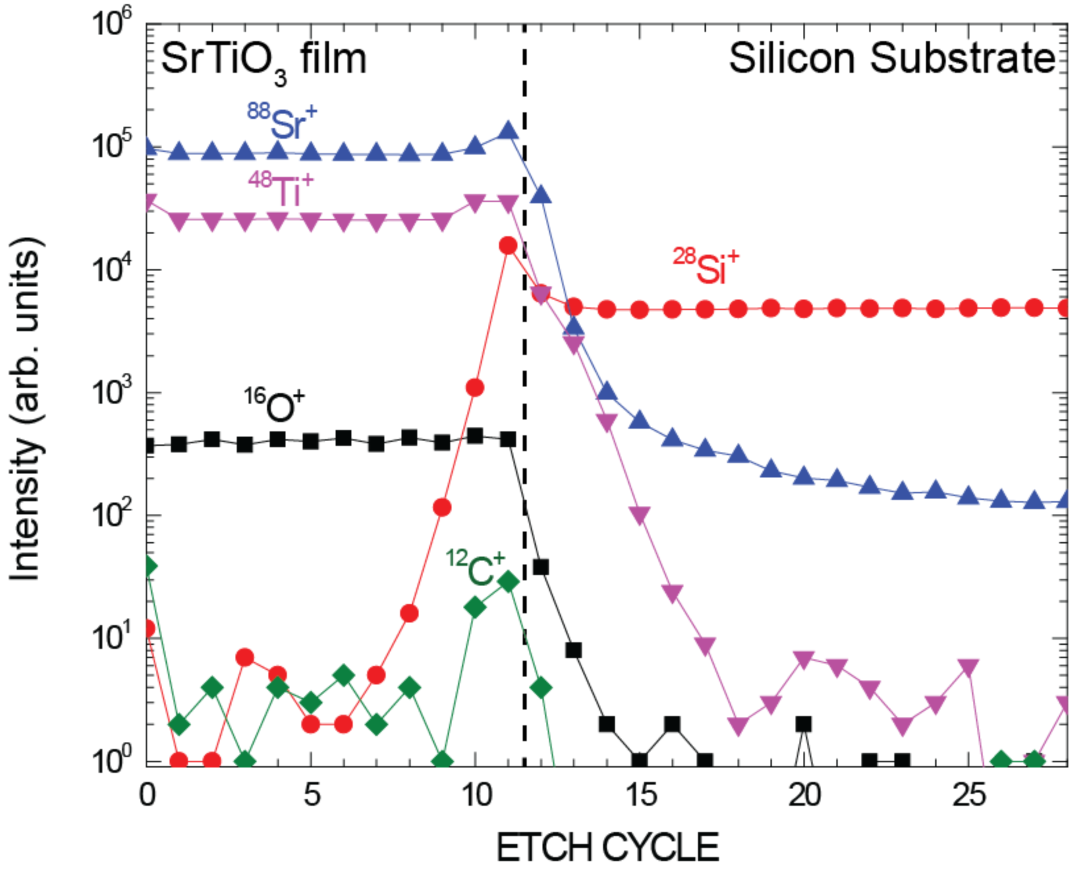

Supplementary References:

1. Lee, H. N., Ambrose Seo, S. S., Choi, W. S. & Rouleau, C. M. Growth control of oxygen stoichiometry in homoepitaxial $SrTiO_3$ films by pulsed laser epitaxy in high vacuum. *Sci. Rep.* **6**, 19941

2. Tomio, T., Miki, H., Tabata, H., Kawai, T. & Kawai, S. Control of electrical conductivity in laser deposited $SrTiO_3$ thin films with Nb doping. *J. Appl. Phys.* **76**, 5886–5890 (1994).

3. Khodan, A. N. *et al.* Pulsed Laser Deposition of epitaxial SrTiO3 films: Growth,structure and functional properties. *Thin Solid Films* **515**, 6422–6432 (2007).

4. Tarsa, E. J., Hachfeld, E. A., Quinlan, F. T., Speck, J. S. & Eddy, M. Growth-related stress and surface morphology in homoepitaxial SrTiO3 films. *Appl. Phys. Lett.* **68**, 490–492 (1996).

5. Ambwani, P. *et al.* Defects, stoichiometry, and electronic transport in $SrTiO_{3-\delta}$ epilayers: A high pressure oxygen sputter deposition study. *J. Appl. Phys.* **120**, 055704 (2016).

6. Taylor, T. R. *et al.* Influence of stoichiometry on the dielectric properties of sputtered strontium titanate thin films. *J. Appl. Phys.* **94**, 3390–3396 (2003).

7. Fuchs, D. *et al.* Structural properties of slightly off-stoichiometric homoepitaxial $SrTixO_{3-\delta}$ thin films. *J. Appl. Phys.* (2000).

8. Goldenberg, E. *et al.* Effect of O2/Ar flow ratio and post-deposition annealing on the structural, optical and electrical characteristics of $SrTiO_3$ thin films deposited by RF sputtering at room temperature. *Thin Solid Films* **590**, 193–199 (2015).

9. Ambwani, P. Transport and Magnetism in Bulk and Thin Film Strontium Titanate. (University of Minnesota, 2015).

10. Yamaguchi, H. *et al.* Structural and Electrical Characterization of $SrTiO_3$ Thin Films Prepared by Metal Organic Chemical Vapor Deposition. *Jpn. J. Appl. Phys.* **32**, 4069–4073 (1993).

11. Gilbert, S. R., Wessels, B. W., Studebaker, D. B. & Marks, T. J. Epitaxial growth of $SrTiO_3$ thin films by metalorganic chemical vapor deposition. *Appl. Phys. Lett.* **66**, 3298–3300 (1995).

12. Fröhlich, K. *et al.* Growth of $SrTiO_3$ thin epitaxial films by aerosol MOCVD. *Thin Solid Films* **260**, 187–191 (1995).

13. Wang, Z. & Oda, S. Atomic Layer-by-Layer Metal-Organic Chemical Vapor Deposition of $SrTiO_3$ Films with a Very Smooth Surface. *Jpn. J. Appl. Phys.* **37**, 942–947 (1998).

14. Lee, S. W. *et al.* Atomic Layer Deposition of $SrTiO_3$ Thin Films with Highly Enhanced Growth Rate for Ultrahigh Density Capacitors. *Chem. Mater.* **23**, 2227–2236 (2011).

15. Vehkamäki, M., Hatanpää, T., Hänninen, T., Ritala, M. & Leskelä, M. Growth of $SrTiO_3$ and $BaTiO_3$ Thin Films by Atomic Layer Deposition. *Electrochem. Solid-State Lett.* **2**,

504 (1999).

16. Vehkamäki, M. *et al.* Atomic Layer Deposition of $SrTiO_3$ Thin Films from a Novel Strontium Precursor-Strontium-bis(tri-isopropyl cyclopentadienyl). *Chem. Vap. Depos.* **7**, 75–80 (2001).

17. Fisher, P. *et al.* Stoichiometric, nonstoichiometric, and locally nonstoichiometric $SrTiO_3$ films grown by molecular beam epitaxy. *J. Appl. Phys.* **103**, 013519 (2008).

18. Yamaguchi, H., Matsubara, S. & Miyasaka, Y. Reactive Coevaporation Synthesis and Characterization of $SrTiO_3$ Thin Films. *Jpn. J. Appl. Phys.* **30**, 2197–2199 (1991).

19. Brooks, C. M. *et al.* Growth of homoepitaxial $SrTiO_3$ thin films by molecular-beam epitaxy. *Appl. Phys. Lett.* **94**, 162905 (2009).

20. Gu, X. *et al.* Growth, characterization, and uniformity analysis of 200 mm wafer-scale $SrTiO_3$/Si. *J. Vac. Sci. Technol. B, Nanotechnol. Microelectron. Mater. Process. Meas. Phenom.* **28**, C3A12-C3A16 (2010).

21. Zhang, L. *et al.* Continuously Tuning Epitaxial Strains by Thermal Mismatch. *ACS Nano* **12**, 1306–1312 (2018).

22. Son, J. *et al.* Epitaxial SrTiO3 films with electron mobilities exceeding 30,000 $cm^2 V^{-1} s^{-1}$. *Nat. Mater.* **9**, 482–484 (2010).

23. Jalan, B., Engel-Herbert, R., Wright, N. J. & Stemmer, S. Growth of high-quality $SrTiO_3$ films using a hybrid molecular beam epitaxy approach. *J. Vac. Sci. Technol. A Vacuum, Surfaces, Film.* **27**, 461 (2009).

24. Brooks, C. M. *et al.* Tuning thermal conductivity in homoepitaxial $SrTiO_3$ films via defects. *Appl. Phys. Lett.* **107**, 051902 (2015).

25. Wang, T., Ganguly, K., Marshall, P., Xu, P. & Jalan, B. Critical thickness and strain relaxation in molecular beam epitaxy-grown $SrTiO_3$ films. *Appl. Phys. Lett.* **103**, 212904 (2013).

26. Björck, M., Andersson, G. & IUCr. GenX: an extensible X-ray reflectivity refinement program utilizing differential evolution. *J. Appl. Crystallogr.* **40**, 1174–1178 (2007).

27. Jalan, B., Cagnon, J., Mates, T. E. & Stemmer, S. Analysis of carbon in $SrTiO_3$ grown by hybrid molecular beam epitaxy. *J. Vac. Sci. Technol. A Vacuum, Surfaces, Film.* **27**, 1365–1368 (2009).
